# Community Perceptions of Air Pollution and Related Health Risks in Nairobi Slums

**DOI:** 10.3390/ijerph10104851

**Published:** 2013-10-11

**Authors:** Thaddaeus Egondi, Catherine Kyobutungi, Nawi Ng, Kanyiva Muindi, Samuel Oti, Steven van de Vijver, Remare Ettarh, Joacim Rocklöv

**Affiliations:** 1 African Population and Health Research Center, P.O. Box 10787, Nairobi 00100, Kenya; E-Mails: ckyobutungi@aphrc.org (C.K.); kmuindi@aphrc.org (K.M.); soti@aphrc.org (S.O.); svijver@gmail.com (S.V.); 2 Department of Public Health and Clinical Medicine, Epidemiology and Global Health, Umeå University, Umeå SE-901 85, Sweden; E-Mails: Nawi.Ng@epiph.umu.se (N.N.); joacim.rocklov@envmed.umu.se (J.R.); 3 Department of Global Health, Academic Medical Centre, University of Amsterdam, Amsterdam Institute for Global Health and Development, Amsterdam 1100 DE, The Netherlands; 4 Faculty of Medicine, University of British Columbia, Vancouver, BC V6T 1Z3, Canada; E-Mail: rettarh@gmail.com

**Keywords:** perceived air quality, air pollution, perceived health risk, urban slum

## Abstract

Air pollution is among the leading global risks for mortality and responsible for increasing risk for chronic diseases. Community perceptions on exposure are critical in determining people’s response and acceptance of related policies. Therefore, understanding people’ perception is critical in informing the design of appropriate intervention measures. The aim of this paper was to establish levels and associations between perceived pollution and health risk perception among slum residents. A cross-sectional study of 5,317 individuals aged 35+ years was conducted in two slums of Nairobi. Association of perceived score and individual characteristics was assessed using linear regression. Spatial variation in the perceived levels was determined through hot spot analysis using ArcGIS. The average perceived air pollution level was higher among residents in Viwandani compared to those in Korogocho. Perceived air pollution level was positively associated with perceived health risks. The majority of respondents were exposed to air pollution in their place of work with 66% exposed to at least two sources of air pollution. Less than 20% of the respondents in both areas mentioned sources related to indoor pollution. The perceived air pollution level and related health risks in the study community were lowamong the residents indicating the need for promoting awareness on air pollution sources and related health risks.

## 1. Introduction

Exposure to urban air pollution is one of several environmental and public health concerns currently confronting the World’s population [1]. Several studies have established an association between air pollution and health effects [2,3,4]. Exposure to air pollution leads to adverse health effects ranging from respiratory illness to chronic illness such as cancer, adverse pregnancy outcomes and premature death. About 3.3 million premature deaths are attributed to both indoor and outdoor air pollution yearly and the burden is high among those living in middle-income countries [5]. Low socioeconomic status generally is associated with poor health, which makes people susceptible to the damaging effects of air pollution [6,7]. Moreover, people with low socioeconomic status such as those living in urban slums are more likely to live closer to roadways and polluting industrial facilities thus exposing them to greater levels of pollutants. They also have less access to health care thereby exacerbating any adverse health outcomes [6]. 

Sources of outdoor air pollution in urban slums are mainly dust, burning of trash, vehicle and industrial emissions. Due to poor ventilation in these settings, outdoor air pollutants infiltrate into households raising levels of indoor air pollution. This combination of indoor and outdoor air pollution increases the burden of air pollution in deprived urban areas. In developing countries, indoor air pollutants are mainly released during the combustion of solid fuels used for cooking and heating. Households using such fuels are generally located in poor communities in rural areas and urban slums with poorly ventilated houses [8]. Though several studies have shown the link between exposure to air pollution and health, most of these studies have been carried out in high-income settings. In addition, few studies have tried to understand the perceptions of residents about air pollution and related risks particularly among the very poor population.

Benefits of reducing exposure to air pollution are well documented from both natural experiments [4,9,10] and epidemiological studies [11,12]. In an effort to reduce air pollution, most air quality management bodies have focused on the emissions-based control programs [13]. For example in Kenya, a draft air pollution regulation set up by the National Environmental Management Authority (NEMA) focuses on controlling the emissions [14]. Although regulations targeting emissions have led to a decrease in levels of pollution, interventions targeting individuals, which reduce exposure independent of emissions, will greatly mitigate health impacts of air pollution [13,15,16,17]. A recent study suggests a new framework of reducing air pollution-related health impacts that incorporates strategies at regulatory, community, and individual levels to reduce both emission and exposure [13]. Strategies targeting either the community or individual levels require contextual knowledge of the perceptions of both exposure and associated risk.

Perception is an important component of behavior change and plays a major role in public response to environmental exposures [18,19,20]. Therefore, increasing people’s perception and knowledge is a cornerstone for interventions promoting protective behavior. This paper adopted the definition of perception by Sjöberg *et al.* as subjective assessment of exposure level to an environmental hazard and the concern with the consequences of the exposure [21]. Research on environmental risk assessment has established a relationship between exposure and health risks. However, little attention has been paid to understanding community perceptions of environmental risk particularly in sub-Saharan Africa. Consequently, governments are grappling with how to empower citizens to be involved in various aspects of environmental management to increase action and local participation in interventions [22].

Furthermore, community and individual level interventions for reducing exposure to air pollution are important means for improving public health and citizen participation. However, the effectiveness of these programs depends mainly on peoples’ perceptions of exposure and risk for individual acceptance and action. Studies on risk perception have revealed it to be multi-dimensional with demographic, cultural and political characteristics playing a role in observed differences in perception.

Annoyance has also been identified as one consequence of air pollution [23] and is said to be a useful signal for potential health effects of pollution in a community [24]. Studies have found that annoyance involves individual perceptions and attitudes towards the exposure [23] influenced by factors such as sex, age and prior exposure to the pollutant [25]. Understanding perception and attitudes, including annoyance, of the public towards air quality and related health risks is therefore critical in informing the design of intervention programs as well as for successful individual involvement in the mitigation process. Interventions directed at individuals have also been suggested to lower baseline health risks and overall burden of diseases associated with air pollution [13,26]. Such interventions include, but are not limited to, control of hypertension and smoking cessation.

### Study Context

The study is set in two informal settlements or slums (Korogocho and Viwandani) located in Nairobi, the capital of Kenya. The two slums form part of the Nairobi Urban Health and Demographic Surveillance System (NUHDSS) run by African Population and Health Research Center (APHRC). Low household incomes, low levels of education, and high proportions of the population employed in manual/unskilled occupations typify both slums. Both areas are characterized by unpaved roads, open burning of refuse and cooking along the road using sawdust or sacks made from sisal or cotton/plastic materials. There are refuse-dumping sites located close to each of these study areas and specifically, the largest refuse dump site in Nairobi is situated to the east and south east of the Korogocho slum settlement. Smoke from these dumping sites bellows out daily, day and night, and even during the rainy season. Viwandani, one of the informal settlements, is located along a line of known polluting industries in the city’s industrial area. Despite lack of actual measurements of air pollution, these characteristics suggest that it is an environmentally disadvantaged neighborhood in terms of air pollution.

Work on the burden of disease in the informal settlements of Nairobi indicates high prevalence of respiratory illness, asthma [27], and acute respiratory infections as the leading contributor to the mortality burden [28] among children. Studies in the two areas have also shown seasonal mortality among under-five children with a high peak during the cold season [29,30,31]. It is postulated that high mortality during the cold season may be due to increase in level of indoor pollution as a result of people trying to keep warm using solid fuels. Despite the apparent numerous sources of both outdoor and indoor air pollution, it remains unclear to what extent slum residents perceive air pollution as a major problem for their health. Therefore, the aims of this study were to establish perceived air pollution levels, health risk and annoyance; investigate the socio-demographic factors associated with perceived air pollution levels and related health risk, and determine the spatial variation in the perceived level of pollution and health risk among slum residents in Nairobi.

## 2. Methods

### 2.1. Study Design and Procedures

Data collection for this study was nested in a larger interventional study within the NUHDSS known as the *Sustainable model for Cardiovascular Health by Adjusting Lifestyle and Treatment with Economic Perspective in settings of Urban Poverty* (SCALE UP) study [32]. The target sample for the SCALE UP study included adults aged 35 years and above living in the two slums of Korogocho and Viwandani that gave informed consent to participate in that study. For its own purposes, participants in the SCALE UP study were recruited through different methods in either slum. In Korogocho, it was done through a census of all adults older than 35 years while in Viwandani, it was done through a random sample of similarly aged residents. The different sampling methods in two areas are not expected to affect the results since the random sample was a representative sample of Viwandani. The basis for the recruitment was the sampling frame of the NUHDSS which has a complete listing of all households and individuals within the Demographic Surveillance Area of the two settlements and is described in detail elsewhere [33].

Our study leveraged the data collection platform of the SCALE UP study to administer our own data collection tools to each consenting participant in both slums. The informed consent form clearly explained to the participants that both studies had separate objectives and elaborated on what these objectives were. Data were collected by trained interviewers who administered a structured questionnaire to participants. The questionnaire asked participants about their perception of air quality, air pollution-related health effects, annoyance with air pollution and sources of information on air pollution in addition to individual characteristics.

### 2.2. Description of Measures

The questionnaire included several questions on air pollution level, perceived associated health risk and annoyance from air pollution as summarized in Table 1. The questions included options of yes/no and a five-point ordinal scale. To measure level of annoyance, the respondents were asked to assume that people’s level of annoyance due to indoor and outdoor air pollution from any source could be stacked on a ladder or staircases of five steps, with low level (1) representing “No Annoyance” and high level (5) representing “Extreme Annoyance”. Then respondents were asked to place themselves on the ladder that corresponds to their level of annoyance due to outdoor or indoor air pollution. The questions were adopted from studies that used similar scales [34,35] though we reduced scales from 11-point scales to 5-point scales. The composite measures were generated using the *alpha* STATA command. This command standardizes each variable to a scale of mean 0 and variance of 1 before combining them into a single score. The final composite measure was obtained by averaging standardized individual items for each participant. The standardized scores generated were then transformed to a scale of 0 to 100 for ease of interpretation. The approach allows combining questions with different response scales. High scores represent perceived high level of air pollution or perceived high health risk associated with air pollution. 

**Table 1:** List of questions for the composite indices of perceived level of pollution and perceived related health risk.

Perceived air pollution		
		How would you rate the quality of air in the community where you live (Viwandani/Korogocho)? Would you say it is (Very Low, Low, High, Very High)
		How would you rate the quality of air in your house? Options (Very Low, Low, High, Very High)
		Which of the following would you say are the sources of outdoor or indoor air pollution within Korogocho/Viwandani (Dust, Vehicle emissions, Industrial emissions, Cooking fuels, Burning trash, Smelly sewage, Cigarette smoking, Other sources)? Options (Yes or No)
		How severe would you say is air pollution in Korogocho/Viwandani from (Dust, Vehicle emissions, Industrial emissions, Cooking fuels, Burning trash, Smelly sewage, Cigarette smoking, Other sources)? Options (None, Low, Moderate, High and Very High)
**Perceived health risks **		
	How much health risk do you think each of the following poses to you and your family (dust, vehicle emissions, industrial emissions, cooking fuels, burning trash, smelly sewage, cigarette smoking, and other sources)? Options (None, Low, Moderate, High and Very High)	
	What health problems do you think are brought about by air pollution (cough/cold, difficulty breathing, eye problems, asthma, cancer, heart problems, headache, other)? Options (Yes/No)	

Individual characteristic variables included sex, age, and duration of stay in the community, marital status, education level and current occupation status. Occupation status was grouped into four main categories namely those involved in business activities, informal employment, formal employment and those not working or involved in agricultural activities. The variable daily work locations exposed to air pollution was defined as the number of daily work locations (next to busy roads, near cooking places, dusty place, and factory) mentioned that are considered highly polluted areas. The variable of information on air pollution used for regression analysis was defined as either heard or never heard any information on air pollution.

### 2.3. Statistical Analysis Approach

The responses to different questions were summarized in terms of proportions by the two study sites. The composite measures were summarized in terms of averages by demographic characteristics to assess the distribution of the perception levels across key characteristics. The differences or association of composite measures with different characteristics were assessed using linear regression analysis. Regression analysis was done in two steps. First, bivariate analysis was performed to assess the association of each factor with perceived level of pollution or health risk independently. The purpose of bivariate analysis was to determine the independent relationship between each of the characteristics and the outcome without controlling for others. Secondly, multiple regression analysis was conducted to assess the association of different factors controlling for the other factors. Regression assumptions were assessed using graphical inspection of residuals from a multiple regression model plotted against fitted values.

Spatial variability in the perceived level of air pollution and related health risks was assessed and visualized using hot spot analysis in ArcGIS. The hot spot analysis tool calculates the Getis-Ord statistic for each geo-referenced household to identify where those households with either high or low attribute values cluster spatially. The analysis looks at each household within the context of neighboring households. A household is considered a statistically significant hot spot if it has a high value and is surrounded by other features with high values as well. The same applies for the households classified as cold spots. In this study, hot spots are areas of households of people with high perceived level of air pollution whereas cold spots are of those households of people with low perceived level of air pollution.

## 3. Results

### 3.1. Description of the Sample

The study was conducted from August to December 2012 and a total sample of 5,317 residents was interviewed (3,887 in Korogocho and 1,430 in Viwandani). The sample distribution by socio-demographic characteristics is summarized in Table 2. In Viwandani, there were more male respondents and this is because the area attracts mainly the population seeking employment in the industries. The two study areas differed in age distribution with Viwandani consisting of younger population compared to Korogocho. About 49% of the residents in Korogocho had lived there for at least 20 years compared to about 16% in Viwandani. Most respondents were married in both study areas with high proportion of respondents not married in Viwandani (30%) compared to Korogocho (15%). The proportion of respondents who never went to school was higher in Korogocho (17%) as compared to Viwandani (4%) and very few respondents had attained college or higher level of education (3% in Viwandani and 1% in Korogocho). The two main occupations of the residents in the two sites were informal business and casual labor.

**Table 2:** Percentage distribution of the study sample by the two study sites.

	Viwandani	Korogocho
	(n = 1,430)	(n = 3,887)
Sex (*χ*^2^(1) = 71.6; *p-*value = 0.000)		
Female	36.1	49.1
Male	63.9	50.9
Respondent’s Age (*χ*^2^(3) = 238.9; *p-*value = 0.000)		
35–40 years	41.9	26.3
41–50 years	41.5	37.7
51–60 years	13.3	21.4
60+ years	3.3	14.6
Duration of stay (*χ*^2^(3) = 595.7; *p-*value = 0.000)		
0–10 years	41.0	17.5
11–20 years	43.3	33.9
21–30 years	14.4	30.9
30+ years	1.2	17.8
Marital status (*χ*^2^(1) = 119.2; *p-*value = 0.000)		
Married	85.3	70.5
Not married	14.7	29.5
Education level (*χ*^2^(2) = 343.5; *p-*value = 0.000)		
Less than primary	15.2	38.0
Primary	50.8	46.2
Secondary+	34.0	15.8
Current Occupation (*χ*^2^(3) = 662.9; *p-*value = 0.000)		
Business	29.4	43.0
Informal	35.1	34.5
Formal	27.0	3.9
Other	8.6	18.6

### 3.2. Location of Work, Sources of Pollution, Health risks and Information on Air Pollution

The findings regarding location of work, health risks, sources of pollution and information on air pollution are summarized in Table 3. Majority of respondents in both study areas reported that their daily work is located next to either a busy road or in a dusty place. In both sites, about half of the respondents reported working near a place where cooking takes place. In Viwandani: 13% of respondents reported working in all the four areas; next to a busy road, in a dusty place, near a place where cooking is taking place and in a factory where air is bad compared to only 3% in Korogocho. In both locations, most of respondents (81%) reported smelly trenches as the source of air pollution, although this is not scientifically considered a source of air pollution. Dust and burning of trash was mentioned as source of air pollution, more often among the respondents in Korogocho than those in Viwandani. Industrial emissions were reported to be a major source of pollution in Viwandani (55%) compared with only 5% in Korogocho. Vehicles were mentioned as a source of pollution by more respondents in Korogocho (20%) compared to Viwandani (7%). 

**Table 3:** The percentage distribution of respondents’ location of work, perceived sources of air pollution and health risks, and sources of information on air pollution by study site.

	Viwandani	Korogocho	Total
	%	%	*p-*value
**Location of respondent’s daily work**			
Next to a busy road	72.0	68.1	0.018
Near a place where cooking takes place	46.9	52.8	0.001
In a dusty place	70.2	68.4	0.260
In a factory where air is bad	40.6	10.4	<0.001
Not in a fixed place	56.4	38.1	<0.001
At least one of the above	71.3	68.6	0.058
**Perceived sources of air pollution**			
*Outdoor air pollution sources*			
Dust	47.5	63.0	<0.001
Vehicles	7.7	20.3	<0.001
Industries	55.5	5.0	<0.001
Burning of Trash	50.5	66.6	<0.001
*Indoor air pollution sources*			
Cooking fuels	13.8	22.1	<0.001
Cigarette Smoking	11.6	18.5	<0.001
*Other*			
Smelling of Trenches	81.1	81.8	0.568
Other sources	9.5	13.1	<0.001
**Perceived health risks from air pollution**			
Cough/Cold	61.5	74.4	<0.001
Difficulty breathing	39.9	46.4	<0.001
Eye problem	15.2	27.7	<0.001
Asthma	10.2	14.7	<0.001
Cancer	6.4	6.5	0.904
Heart problem	15.1	13.3	0.089
Headache	16.5	30.1	<0.001
Don’t know	10.2	6.3	<0.001
OTHER health risks	24.5	17.6	<0.001
**Sources of information on air pollution**			
Radio	32.3	39.1	<0.001
TV	12.5	14.6	0.059
Newspapers	5.7	6.1	0.629
Barazas (Community meetings)	5.4	18.4	<0.001
Health workers/facilities	9.7	25.1	<0.001
Never heard	55.0	35.1	<0.001

The two main sources of indoor air pollution being cooking fuels and cigarette smoking were among the least reported source of air pollution in Viwandani. Cough/cold, difficulties in breathing, headache and eye problems were the most common health risks mentioned related to air pollution. Only 10% of the respondents in Viwandani and 6% in Korogocho did not know of any health problems related to air pollution. Radio was reported as main source of information regarding air pollution though 55% of respondents in Viwandani and 35% in Korogocho had never received any information related to air pollution.

### 3.3. Factors Related to Community Perceptions on Air Pollution

The average score of perceived air pollution level was 46.9 (95% CI = 46.0 to 47.8) in Viwandani and 41.4 (95% CI = 40.9 to 41.9) in Korogocho. The average score for perceived level of health risk related to air pollution was 43.6 (95% CI = 42.7 to 44.5) in Viwandani and 44.6 (95% CI = 44.1 to 45.1) in Korogocho. Descriptive summary statistics for the perceived scores are presented in Table 4. 

**Table 4:** Descriptive summary of the perceived scores by individual characteristics

	Air pollution perceived score Mean (SD)	Health risk perceived score Mean (SD)	Annoyance Mean (SD)
*Study Site*			
Viwandani	46.9 (17.3)	43.6 (17.4)	41.7 (18.8)
Korogocho	41.4 (15.8)	44.6 (16.6)	48.3 (18.2)
*Sex*			
Female	42.5 (16.1)	44.1 (16.9)	46.8 (18.6)
Male	43.2 (16.7)	44.5 (16.8)	46.2 (18.7)
*Respondent’s Age*			
35–40 years	44.1 (17.0)	44.8 (16.7)	46.3 (18.3)
41–50 years	43.1 (16.4)	44.5 (16.9)	46.6 (18.9)
51–60 years	41.8 (16.0)	44.0 (16.7)	46.7 (18.7)
60+ years	40.7 (15.5)	43.1 (17.0)	46.4 (18.3)
*Duration of Stay*			
0–10 years	44.3 (16.8)	44.1 (16.5)	45.6 (18.9)
11–20 years	43.2 (16.8)	44.4 (17.2)	46.8 (18.2)
21–30 years	42.4 (15.8)	44.1 (16.5)	46.4 (18.6)
30+ years	40.6 (15.6)	45.1 (17.0)	47.5 (19.2)
*Marital status*			
Married	42.2 (15.7)	43.8 (16.4)	47.3 (18.4)
Not married	43.3 (16.5)	44.7 (16.7)	46.2 (18.7)
*Education Level*			
Less than Primary	40.9 (15.2)	42.7 (17.2)	45.5 (19.1)
Primary	43.8 (16.7)	45.5 (16.3)	47.1 (18.0)
Secondary+	44.4 (16.9)	45.0 (16.5)	46.6 (19.2)
*Current Occupation*			
Business	42.2 (16.3)	45.1 (16.4)	46.8 (18.2)
Informal	44.0 (16.6)	45.4 (16.6)	47.2 (18.7)
Formal	44.2 (16.4)	41.1 (16.4)	43.1 (19.2)
Other	42.2 (15.9)	43.0 (17.1)	46.4 (19.0)
**Total**	**42.9 (16.4)**	**44.3 (16.8)**	**46.5 (18.6)**

At bivariate analysis, perceived air pollution was associated with duration of stay in the slum, age of the respondent, education level, occupation, received information on air pollution, number of work locations exposed to air pollution and slum of residence. Respondents who had stayed longer in the study area or those who were older perceived low level of air pollution when considered independently. However, this association was not significant after controlling for other factors. Residents in Korogocho had perceptions of low air pollution level compared to residents in Viwandani in the bivariate analysis. Therefore, final regression analysis for both perceived level of air pollution and health risk was performed for each slum of residence separately.

The results of the multiple linear regressions by study site are shown in Table 5. Duration of stay, age, gender and marital status were not significantly associated with perceived air pollution level in analysis stratified by study site. 

**Table 5:** Characteristics associated with perceived level of air pollution and related health risks.

	Air pollution		Health Risk	
	Viwandani	Korogocho	Viwandani	Korogocho
Perceived pollution level			0.44 *	0.48 *
Duration stay (ref: –10 years)				
11–20 years	−1.16	0.63	1.32	−0.47
21–30 years	−2.17	0.77	2.13	−0.96
30+ years	−3.70	0.09	3.14	1.47
Age (ref: 35–40 years)				
41–50 years	0.46	−0.54	−0.27	0.20
51–60 years	1.11	−1.37	0.28	−0.11
60+ years	4.43	−1.46	2.21	0.20
Male	−0.23	−0.85	−1.86	0.30
Married (ref: Not married)	0.49	−0.20	0.21	1.09 *
Education (ref: No education)				
Primary	3.80 *	0.80	−0.78	1.60 *
Secondary +	3.44 *	0.38	−0.71	1.29
Occupation (ref: Business)				
Informal	−0.50	1.25 *	−0.87	−0.89
Formal	−3.20 *	4.88 *	−1.26	−1.87
Other/None	2.42	5.12 *	3.85 *	−1.50 *
Never heard any information	2.31 *	−2.19 *	−5.06 *	−1.4 *
Number of polluted work locations	9.61 *	5.40 *	10.05 *	0.97
Annoyance level	0.38 *	0.07 *	0.07 *	0.21 *

* Statistically significant at 5% level and the values represent linear regression coefficients.

Individuals with at least primary level of education perceived higher levels of air pollution compared to those with no or less than primary level. However, the relationship between education and perceived air pollution level was statistically significant only among residents of Viwandani. Occupation was significantly associated with perceived air pollution level and the relationship varied by the study site. In Viwandani, people engaged in formal employment perceived lower levels of air pollution and the perception of those in informal or other forms of occupations were not significant compared to those in business. Those in informal, formal and other forms of employment perceived higher levels of air pollution compared to those in business among Korogocho residents. Respondents who never heard information on air pollution significantly perceived lower levels of air pollution in Korogocho. Surprisingly, persons who never heard air pollution information in Viwandani perceived higher levels of air pollution compared to those who had. Exposure to working environments with air pollution was positively associated with perceived air pollution level. Perceived air pollution level was also positively associated with annoyance level among residents in both areas.

Perceived level of health risk was positively associated with perceived air pollution level in both areas. Duration of stay, age and gender were not significantly associated with perceived health risk related to air pollution after controlling for other individual characteristics. Perceived health risk was associated with marital status, education level, and occupation in multivariate analysis. Married people in Korogocho had a higher perceived health risk. Education was significantly associated with high perceived health risk in Korogocho. Other or no occupation was associated with perceived high health risk among residents in Viwandani and low perceived health risk among Korogocho residents. Those who had not heard information about air pollution were associated with low perceived risk in both areas. A similar positive association of highly perceived level of air pollution with exposure to working environments with air pollution and annoyance from air pollution was observed for perceived health risks. 

**Figure 1:**
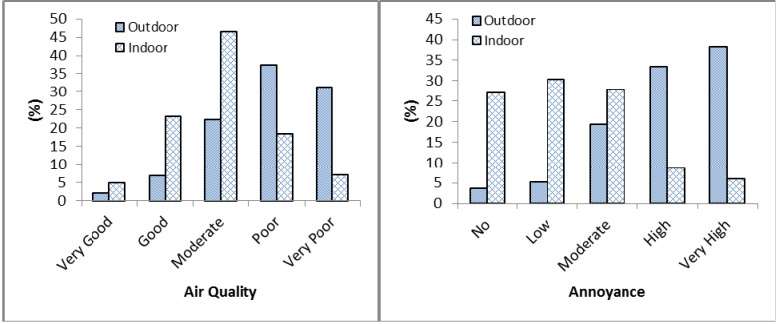
The percentage rating of air quality and the level of annoyance from air pollution for both outdoor and indoor environments.

Outdoor air quality was considered poor compared to indoor air quality. A majority of residents felt that the quality of indoor air was moderate or good and were mostly not annoyed by it. Annoyance level about outdoor air quality may directly relate to the perceived air pollution level. The variation in perceived air quality and annoyance level is illustrated in Figure 1 for Korogocho and Viwandani settlements, respectively.

### 3.4. Spatial Variation in Perceptions

**Figure 2:**
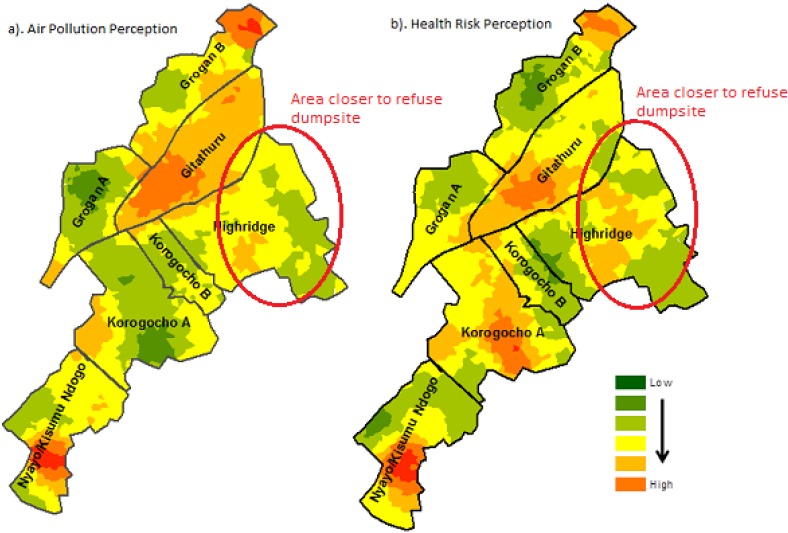
Perceptions on the level of air pollution and related health risks across villages in Korogocho.

**Figure 3:**
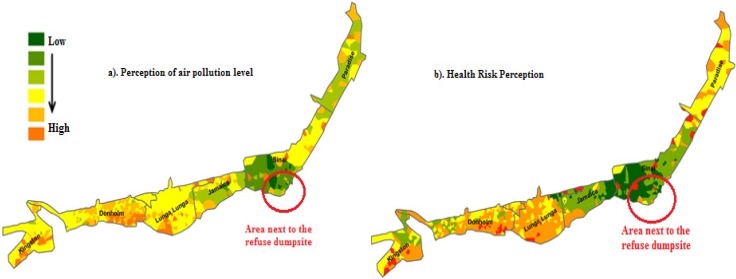
Perceptions on the level of air pollution and related health risks across villages in Viwandani.

Figure 2 and Figure 3 illustrate the distribution of perceived air pollution levels by village across the slums. The darker orange color represents areas in which respondents believe the local air pollution level was high. 

The general spatial pattern of poor air quality perceptions is concentrated in some specific areas similar to perceived health risks in both areas. The spatial pattern particularly in Korogocho seems to be influenced by the residents’ activities and not the location of major sources of air pollution. For example, respondents closer to a major refuse dumpsite (Highridge village) had low knowledge of air pollution despite the constant bellowing of smoke from the dumpsite. A heterogeneous pattern of air quality perceptions was observed in all villages of both slums, representing a spatial mix of views on environmental quality. The spatial patterns observed for both air quality and health risks in the two slums indicate that perceptions of health risks are associated with the perceived air pollution level.

As stated above, Viwandani has a similar spatial pattern of local air quality perceptions where those who expressed high air pollution level were located within villages closer to industries believed to emit pollutants in the air. For example, residents closer to the paper industry in Donholm village believed there was high air pollution level (poor air quality). Poor drainage of wastes from industries particularly in Kingstone and Lunga Lunga areas also made the residents perceive high levels of air pollution in the area. In the central area (Sinai village), residents had low perception of poor air quality despite being closer to a dumpsite, which emits heavy smoke from open fires. The industries close to this area (Sinai) are construction and food production industries that are believed not to emit pollutants in the air as compared to other areas. Thus low perceived air pollution of the residents in this area might be based on this kind of belief. The burning of tires in Paradise village also raises concerns of air quality by the residents. Compared to Korogocho, the patches of perception for Viwandani are clearly distinct though there is heterogeneity with respect to perceptions in both slums. These results illustrated in the two figures also indicate that perceived levels of air pollution were higher in Viwandani compared to Korogocho.

## 4. Discussion

The main goal of this study was to examine perceived air quality, related health risks and associated individual characteristics using a regression analysis. The study also examined spatial variation of the perceived air quality and related risk using spatial analysis. The key findings of this study are as follows; That there is very poor perception of actual sources of air pollution levels and misconception of what ordinarily are not air pollution sources such as “smelly trenches” among residents in the two communities, there is poor perception on the magnitude and importance of indoor air pollution. The majority of the residents also expressed that they were exposed to air pollution in their workplaces.

We found that those respondents who perceived high levels of air pollution in their local community had at least primary level of education or were likely to be in formal employment among residents of the Viwandani area. The difference in the observed relationship between perception and individual characteristics in the two study areas can be explained by the difference in the activities taking place. Among major contributors of air pollution, residents mentioned dust, burning of trash and industries though they were more concerned with smelling of trenches as a result of poor drainage. We also found that there was considerable concern about cooking fuels as source of pollution but few people felt that vehicles were source of air pollution in the two study areas. However, research findings from other settings have shown that people largely perceive automobiles and industries as the major source of pollution [36,37,38].

Several studies have explored the association between respondents’ perceptions and individual characteristics. The relationship between level of education and air quality perception or awareness remains controversial. We found that respondents with at least primary level of education were more concerned about air quality than those with no or less than primary level in Viwandani and the relationship was not significant in Korogocho. Previous studies have reported higher levels of education to be associated with higher annoyance level or poor air quality perceptions [7,39]. Contrary, some studies indicate that a low education level was a significant determinant of annoyance with perceived air pollution level [40,41], a finding that could have been confounded by the place of residence of these people. Socio-economic variables have been shown to be correlated with the perception of local air quality, suggesting that these may be important factors in a study of perceived air quality [42]. In our study, we found that involvement in different forms of occupation was associated with varying perception of poor air quality depending on the location. The importance of socioeconomic indicators in the context of air pollution research has been emphasized because they represent underlying aspects that affect susceptibility, exposure, or disease diagnosis and treatment [6]. Therefore, there is need for careful choice and interpretation of socioeconomic indicators depending on the location.

Though we did not find a significant association between age and perceptions of air quality, a study about the risks of greatest concern found that young respondents were more concerned about environmental issues, while older respondents were more likely to emphasize health and safety [43] while in another study it was found that young people aged 20–34 years had poor air quality perceptions compared to older age groups [7]. Lack of significant association between age and perceived air quality may be due to our study limitation of including only residents aged 35 and above. We did not find significant association between duration of stay in the study area and perceived air quality after adjusting for other individual characteristics. 

In our study, we found that marital status was not associated with perceived air quality though there is relatively little written about any relation between marital status and perception of air quality and environmental issues. However, we observed significant association between marital status and high-perceived health risk in Korogocho. In his study, Kim *et al.* [7] found that ever married people are likely to perceive air quality as poor. Though explanation was not provided for this finding, in literature it is explained that married people may be more concerned about environmental pollution because spouses may be an important influence or source of information on environmental issues [44]. 

After adjusting for individual characteristics, we observed significant association between perceived air quality and perceived health risks related to air pollution. Previous studies reported a significant association between perceived air quality and self-reported health status [45,46]. Though self-reported health and perceived health risk refers to different concepts. Perceived health risk related to air pollution was found to be associated with level of education and the type of occupation though it varied by study location. Those in formal employment perceived low health risk as compared to those involved in business among Viwandani residence while opposite was observed in Korogocho. The fact that most business activities in the slum settlements are mainly informal and carried out along the roads or areas that are associated with high level of pollution can explain this observation. The difference between the two sites could be explained by the difference in the education levels of the residents. There were more people with no education in Korogocho as compared to Viwandani.

It is important to note that air quality perceptions mark differences in the two slums which indicates that perceptions in general may depend on an area's overall setting and availability of industries, other pollution sources or daily activities. For example, an important result from this study was that respondents living in villages closer to industries or poor drainage were more likely to perceive the air as more polluted than those living in other areas. Nevertheless, it is of concern that residents live in close proximity to some sources of pollution such as refuse dumpsite in both areas and yet do not perceive air pollution as a problem. Literature on perceptions of pollution has shown that people tend to attribute pollution to other areas away from their place of residence [1,42]. This could be the same tendency noted among residents of the villages next to known pollution sources; clearly showing the “halo effect”. The use of GIS to map and spatially analyze the differences of air quality perceptions across the two slums helps in forming a clearer picture of how locations influence perceptions of environmental conditions.

## 5. Conclusions

The results of our study may help policymakers understand the need for education programs aimed at making residents in local neighborhoods aware of sources of air pollution and related health risks. The environmental management authorities need to direct effort on risk communication strategies to motivate personal direct perception or awareness of an environmental problem such as air pollution. This approach enhances the individual's understanding of the importance of environmental policy measures, which makes such measures easier to accept by community residents [47] and also enhance personal response in reducing exposure to pollutants. There is also need for further research on community perceptions to help understand factors shaping people’s perceptions.
